# Enhancing targeted transgene knock‐in by donor recruitment

**DOI:** 10.1111/cpr.13163

**Published:** 2021-12-02

**Authors:** Guanyi Jiao, Chenxin Wang, Yangcan Chen, Moyu Dai, Ying Zhang, Wei Li

**Affiliations:** ^1^ State Key Laboratory of Stem Cell and Reproductive Biology Institute of Zoology Chinese Academy of Sciences Beijing China; ^2^ Institute for Stem Cell and Regenerative Medicine Chinese Academy of Sciences Beijing China; ^3^ University of Chinese Academy of Sciences Beijing China; ^4^ Beijing Institute for Stem Cell and Regenerative Medicine Chinese Academy of Sciences Beijing China

## CONFLICT OF INTEREST

The authors declare no competing interests.

## AUTHOR CONTRIBUTIONS

W.L. and Y.Z. conceived this project and supervised the experiments. G.J., C.W., Y.C. and M.D. performed the experiments. G.J. analysed the data. G.J., C.W., Y.C. and Y.Z. wrote the paper with the help from all authors.

Dear Editor,

With the development of clustered regularly interspaced short palindromic repeats (CRISPR)/Cas9^1^‐based gene editing technology, obtaining gene‐knockout cells or animal models has become increasingly convenient. However, other genomic DNA manipulations, including targeted transgene integration or replacement, remain challenging, especially for the treatment of diseases with gene therapy.

Targeted transgenes knock‐in are achieved using strategies based on intrinsic DNA repair mechanisms such as homologous‐directed recombination (HDR)^2^ and non‐homologous end joining (NHEJ).^3^ Marked efforts have been made to improve the efficacies of these knock‐in strategies. Homology‐mediated end joining (HMEJ)^4^ is reported to increase the efficiency of targeted integration by adding a pair of CRISPR/Cas9 targeting sequences flanking the homologous arms of the HDR donor. Homology‐independent targeted integration (HITI)^5^ elevates the frequencies of intended transgene insertion (but not reverse integration) by elaborate arrangement of the orientations of the protospacers in the NHEJ donor.

**FIGURE 1:**
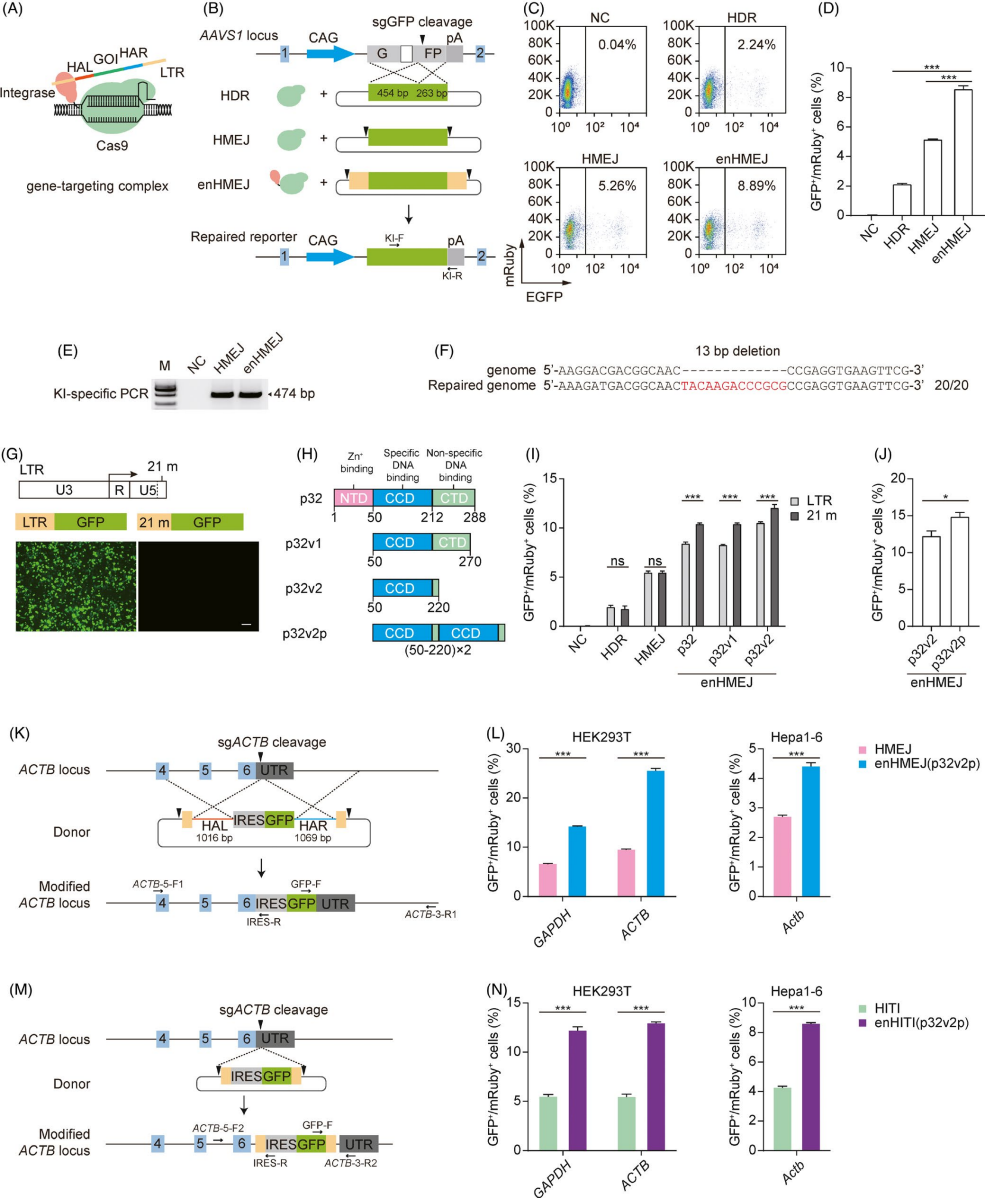
Increasing targeted integration efficiency by donor recruitment. (A) Schematic of the enhanced targeted integration system. The p32‐Cas9/sgRNA complex double‐cleaves the donor vector pPNT6‐LTR‐GOI to generate the LTR‐GOI‐LTR fragment, and p32 protein would bind to the LTR‐GOI‐LTR fragment. The genome is then cleaved by p32‐Cas9/sgRNA, and the LTR‐GOI‐LTR fragment is recruited to the double‐stranded break (DSB) site in the genome. Pink oval indicates integrase p32 of HIV‐1, red box represents the left homologous arm (HAL), blue box represents the right homologous arm (HAR), green box represents the gene of interest (GOI), and yellow box represents LTR. (B) Schematic of the traffic reporter cell line repaired by the truncated EGFP donor based on HDR, HMEJ and enHMEJ. KI‐F/R, knock‐in specific primers. (C) Representative FACS analysis results of the frequencies of EGFP^+^ cell populations. NC, negative control. (D) Statistics of the relative knock‐in efficiency shown in (B). Bars represent the mean + SD of three samples. ***p < 0.001 (unpaired Student's *t* test). (E) Genotyping analysis of the targeted mixed cells aimed at the deleted region. (F) Sequencing analysis of the targeted mixed cells. Red text indicates the repaired sequence. (G) Promoter activity test of LTR and 21 m LTR (21 m). Scale bar, 100 µm. (H) Schematic of the domains of p32 and its variants. (I and J) Statistics of the targeted integration efficiency based on different combinations between endonuclease and modified donor. ***p < 0.001, *p < 0.05, ns, not significant (unpaired Student's *t* test). (K) Schematic of targeted integration based on enHMEJ at the *ACTB* locus. Red and blue lines represent HAL and HAR respectively. (L) Targeted integration efficiency based on HMEJ and enHMEJ (p32v2p/21 m). (M) Schematic of targeted integration based on enHITI at the *ACTB* locus. (N) Targeted integration efficiency based on HITI and enHITI (p32v2p/21 m)

Another technical route to enhance the efficiency of targeted transgene integration is to recruit donor vectors to the targeted genomic site. Some proteins fused to Cas9 can enrich the specific modified single‐stranded oligodeoxynucleotide (ssODN) at the double‐stranded break (DSB) site to increase the targeted integration efficiency.^6^, ^7^, ^8^ However, such systems with chemically modified donor might be unsuitable for delivery in the form of clinically approved viruses such as recombinant adeno‐associated viruses (rAAVs), which drastically restrain the application of these vectors in gene therapies. Here, we present a strategy for enhanced HMEJ (enHMEJ) and enhanced HITI (enHITI) by fusing Cas9 with a specific DNA‐binding protein, integrase p32, from the human immunodeficiency virus type 1 (HIV‐1), to recruit donors harbouring long terminal repeats (LTRs) to the DSB site to increase the targeted integration efficiency *in vivo*. Following transfection of the plasmid compounds pX330‐p32‐Cas9‐CMV‐mRuby2, pPNT6‐LTR‐GOI and pUC19‐U6‐sgRNA into cells, the conjugated protein p32‐Cas9 is produced from the vector pX330‐p32‐Cas9‐CMV‐mRuby2 while single guide RNA (sgRNA) is produced from the vector pUC19‐U6‐sgRNA. The LTR‐flanked transgene is excised from the donor vector by the functional p32‐Cas9/sgRNA complex and is then recruited to the genomic cleavage site mediated by the interaction between LTR and the p32 DNA‐binding domain (Figure 1A and S1).

Initially, we determined whether the p32‐Cas9 hybrid protein could bind the LTR in cells. The results of ChIP‐qPCR show that there was a strong interaction between p32‐Cas9 and LTR (Figure S2). Then to evaluate the contribution of p32/LTRs to the efficiency of HMEJ‐based targeted integration, a 293T‐enhanced green fluorescent inhibited protein (EGIP) cell line was constructed with a 13‐bp deletion in the EGFP coding region driven by a single allelic CAG promoter at the AAVS1 locus (Figure S3). When the deletion is correctly repaired, the 293T‐EGIP cells will express functional EGFP. Thus, the absolute efficiency of gene editing could be assessed by fluorescence‐activated cell sorting (FACS) (Figure 1B).

We found that the knock‐in efficiency of enHMEJ (p32/LTR) was 8.61% ± 0.29%, which was significantly higher than the knock‐in efficiency of canonical HMEJ (5.19% ± 0.06%) and HDR (2.16% ± 0.08%). These results indicated that there was a 1.5‐fold and fourfold increase in targeted integration efficiency for enHMEJ (p32/LTR) compared with HMEJ and HDR respectively (Figure 1C,D). Genotyping analysis of specific missed fragments and sequencing indicated precise gene editing (Figure 1E,F).

For *in vivo* gene integration, any unintended gene expression introduced in the *cis*‐element could be dangerous. The primary sequence of the LTRs we used exhibited promoter activity in 293T cells (Figure 1G). To eliminate this risk, the key 21‐bp‐terminal region of the LTR, which is responsible for binding to integrase, was retained by removing the U3 enhancer and R promoter region.^9^ The resulting engineered 21‐mer LTR (21 m) did not exhibit promoter activity (Figure 1G). On the other hand, the HIV integrase p32 contains an N‐terminal domain (NTD), catalytic core domain (CCD) and C‐terminal domain (CTD). The NTD domain is the Zn^+^ binding domain, which is relative to 3′‐processing and strand transfer reactions. The CCD domain was reported to contain a specific DNA‐binding site, and the CTD domain contains a non‐specific DNA‐binding site.^9^ To address safety concerns, we removed the NTD domain and reassembled the CCD and CTD domains to obtain recombinant protein p32 variant 1 (p32v1) and variant 2 (p32v2; Figure 1H).

To investigate whether these engineered elements could increase the knock‐in efficiency, the construct combinations were transfected into the 293T‐EGIP cell line. Notably, p32v2‐Cas9 exhibited the highest knock‐in efficiency with the 21 m‐modified donor (Figure 1I and S4A). As p32 functions as a dimer, we conjectured that further duplication of p32v2 would enhance the knock‐in efficiency, and congruent with this hypothesis, the engineered p32v2 plus (p32v2p) significantly increased the knock‐in efficiency from 12.13% ± 0.34% to 15.6% ± 0.7% (Figure 1J and S4B). Compared with the canonical HMEJ, the enHMEJ (p32v2p/21 m) system exhibited a threefold increase in gene knock‐in efficiency.

To verify whether the optimized system could increase the efficiency of site‐specific gene integration at endogenous sites, an IRES‐EGFP reporter gene was constructed to target the 3′ untranslated region (UTR) of *ACTB* and *GAPDH* in 293T cells and *Actb* in mouse Hepa1‐6 cells (Figure 1K and S5A). Also, a T2A‐GFP reporter gene was integrated with the final codon of *Sox2* and *Nanog* in mouse embryonic stem cells (Figure S6A). Seven days after the transfection, enHMEJ (p32v2p/21 m) exhibited the same twofold to threefold increase in knock‐in efficiency compared with HMEJ (Figure 1L, S5B and S6B,C). PCR amplification for the integration at the 5′ and 3′ junctions of these targeting sites revealed that the integration based on enHMEJ (p32v2p/21 m) was accurate (Figures S5C,D and S6D,E).

HITI is another site‐specific and homologous arm‐independent targeted integration method. We therefore tested whether our p32v2p/21 m system could enhance HITI efficiency in targeted integration. The 21 m‐modified IRES‐EGFP gene reporter donor was constructed for *ACTB* and *GAPDH* in 293T cells, and *Actb* in mouse Hepa1‐6 cells (Figure 1M and S7A). Seven days after transfection, p32v2p increased the targeted integration efficiency based on HITI by twofold to threefold (Figure 1N and S7B). Genotyping and sequencing analyses showed correct integration of exogenous gene fragments (Figure S7C,D).

In conclusion, we developed a simple system to increase the efficiency of gene integration without the need for any *ex vivo* chemical modification. Importantly, we simplified and optimized our system to erase the potential off‐target risks caused by the promoter activity of the LTR and catalytic activity of the integrase p32. Gene‐targeted cell lines and animal models will be generated more efficiently with the system. Moreover, the high‐efficiency integration *in vivo* indicates a greater curative effect and lower virus injection dose for gene therapy, which is significant for patients so that they receive a safe treatment and regain their health.^10^ Thus, the system has considerable potential for gene therapy applications.

## Supporting information

Supplementary Material

## Data Availability

The data and study materials that support the findings of this study will be available to other researchers from the corresponding authors on reasonable request.

## References

[1] Cong L , Ran FA , Cox D , et al. Multiplex genome engineering using CRISPR/Cas systems. Science. 2013;339(6121):819‐823. 23287718 10.1126/science.1231143 PMC3795411

[2] Yang H , Wang H , Shivalila CS , Cheng AW , Shi L , Jaenisch R . One‐step generation of mice carrying reporter and conditional alleles by CRISPR/Cas‐mediated genome engineering. Cell. 2013;154(6):1370‐1379. 23992847 10.1016/j.cell.2013.08.022 PMC3961003

[3] He X , Tan C , Wang F , et al. Knock‐in of large reporter genes in human cells via CRISPR/Cas9‐induced homology‐dependent and independent DNA repair. Nucleic Acids Res. 2016;44(9):e85. 26850641 10.1093/nar/gkw064 PMC4872082

[4] Yao X , Wang X , Hu X , et al. Homology‐mediated end joining‐based targeted integration using CRISPR/Cas9. Cell Res. 2017;27(6):801‐814. 28524166 10.1038/cr.2017.76 PMC5518881

[5] Suzuki K , Tsunekawa Y , Hernandez‐Benitez R , et al. In vivo genome editing via CRISPR/Cas9 mediated homology‐independent targeted integration. Nature. 2016;540(7631):144‐149. 27851729 10.1038/nature20565 PMC5331785

[6] Carlson‐Stevermer J , Abdeen AA , Kohlenberg L , et al. Assembly of CRISPR ribonucleoproteins with biotinylated oligonucleotides via an RNA aptamer for precise gene editing. Nat Commun. 2017;8(1):1711. 29167458 10.1038/s41467-017-01875-9 PMC5700129

[7] Aird EJ , Lovendahl KN , St Martin A , Harris RS , Gordon WR . Increasing Cas9‐mediated homology‐directed repair efficiency through covalent tethering of DNA repair template. Commun Biol. 2018;1:54. 30271937 10.1038/s42003-018-0054-2 PMC6123678

[8] Savic N , Ringnalda FC , Lindsay H , et al. Covalent linkage of the DNA repair template to the CRISPR‐Cas9 nuclease enhances homology‐directed repair. Elife. 2018;7:e33761. 29809142 10.7554/eLife.33761 PMC6023611

[9] Kessl JJ , McKee CJ , Eidahl JO , Shkriabai N , Katz A , Kvaratskhelia M . HIV‐1 integrase‐DNA recognition mechanisms. Viruses. 2009;1(3):713‐736. 21994566 10.3390/v1030713 PMC3185514

[10] High KA , Roncarolo MG . Gene therapy. N Engl J Med. 2019;381(5):455‐464. 31365802 10.1056/NEJMra1706910

