# Supplementary material for: Enhancing targeted transgene knock‐in by donor recruitment
Source: Cell Prolif. 2021 Dec 2;55(1):e13163. doi: 10.1111/cpr.13163 (PMC8780899; doi:10.1111/cpr.13163)
Supplement: Supplementary file 1 — Supplementary Material [file CPR-55-e13163-s001.docx]

**Supplementary Information**

**Supplementary FigureS AND FIGURE Legends**

**Materials and Methods**

**Supplementary Tables**

**Supplementary FigureS AND FIGURE Legends**


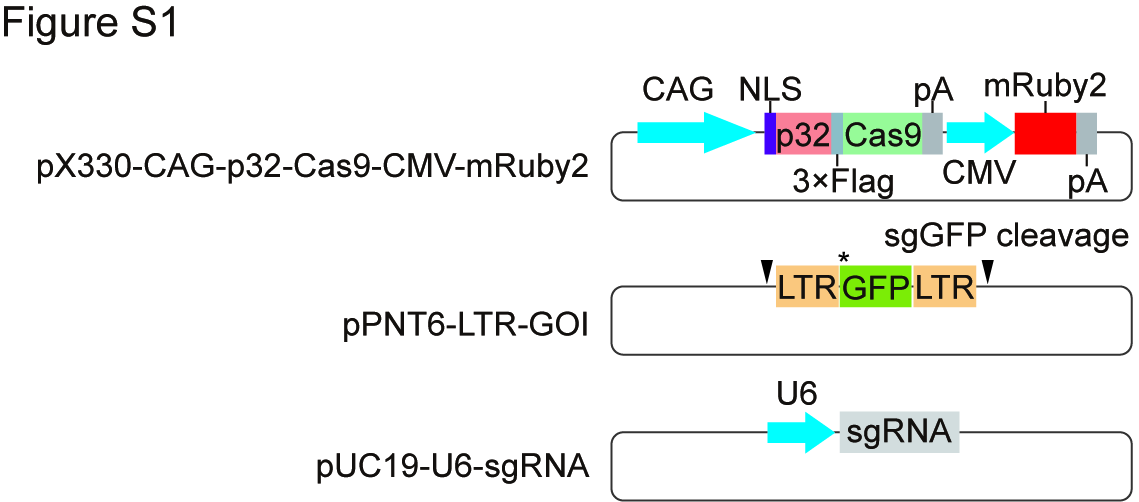


FIGURE S1

Schematic vectors structure of the donor recruitment system in targeted integration. *indicates the start codon “ATG” was deleted in the GFP open reading frame (ORF) to avoid GFP being transcribed by the LTR.


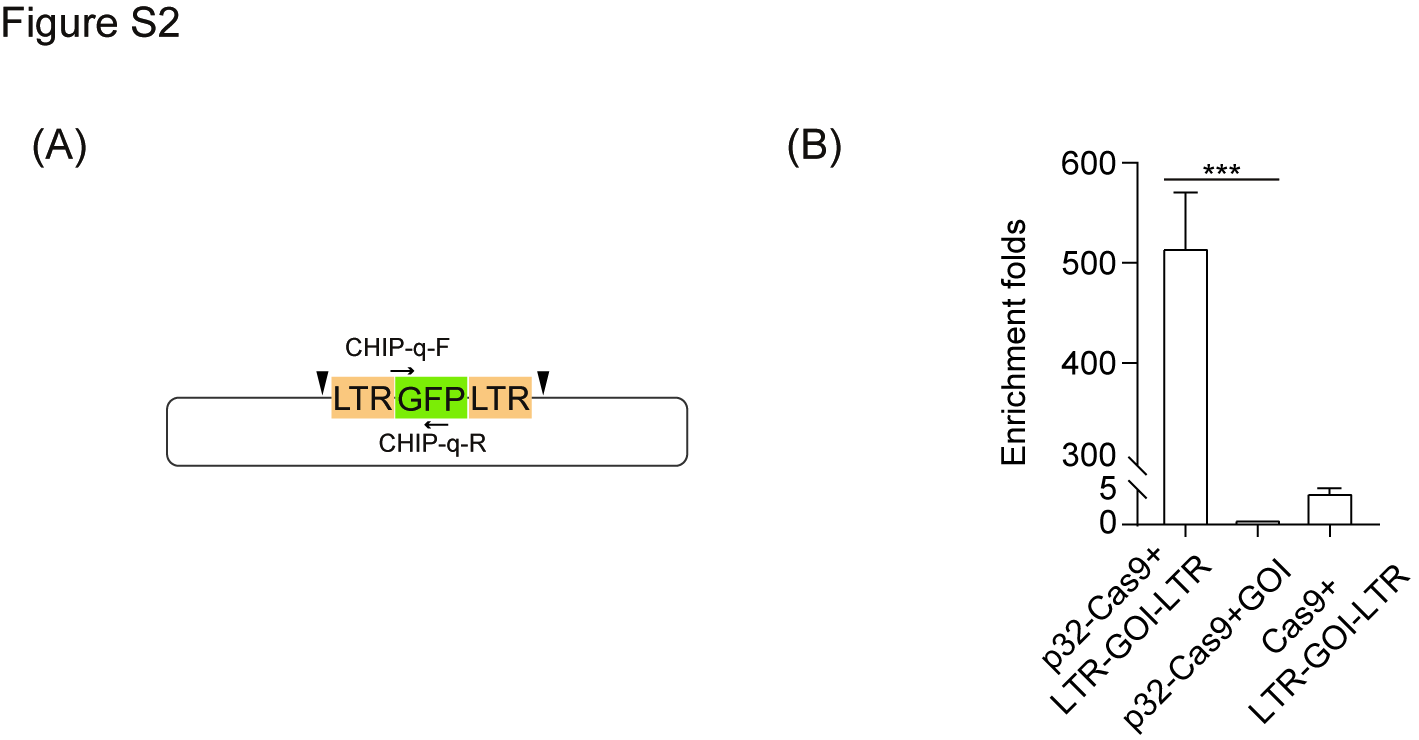


FIGURE S2

Detection of the interaction between p32 and LTR. (A) Schematic structure of pPNT6-LTR-GFP. Black arrows indicate the primer sites used in ChIP-qPCR. (B) ChIP-qPCR results. p32-Cas9+GOI was used as the negative control and the IP efficiency was set as 1. ****P* < 0.001 (unpaired Student’s *t*-test).


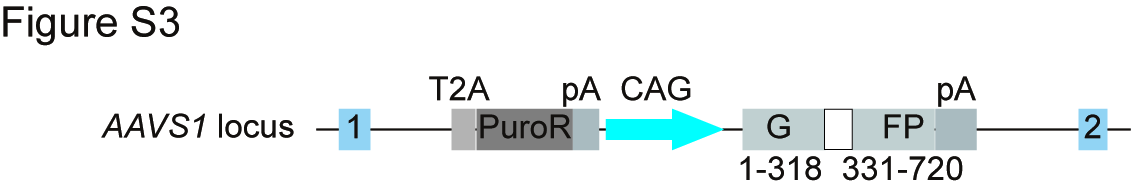


FIGURE S3

Schematic of gene structure of 293T-EGIP cell line. The 13-bp deletion in the open reading frame (ORF) of GFP is shown.


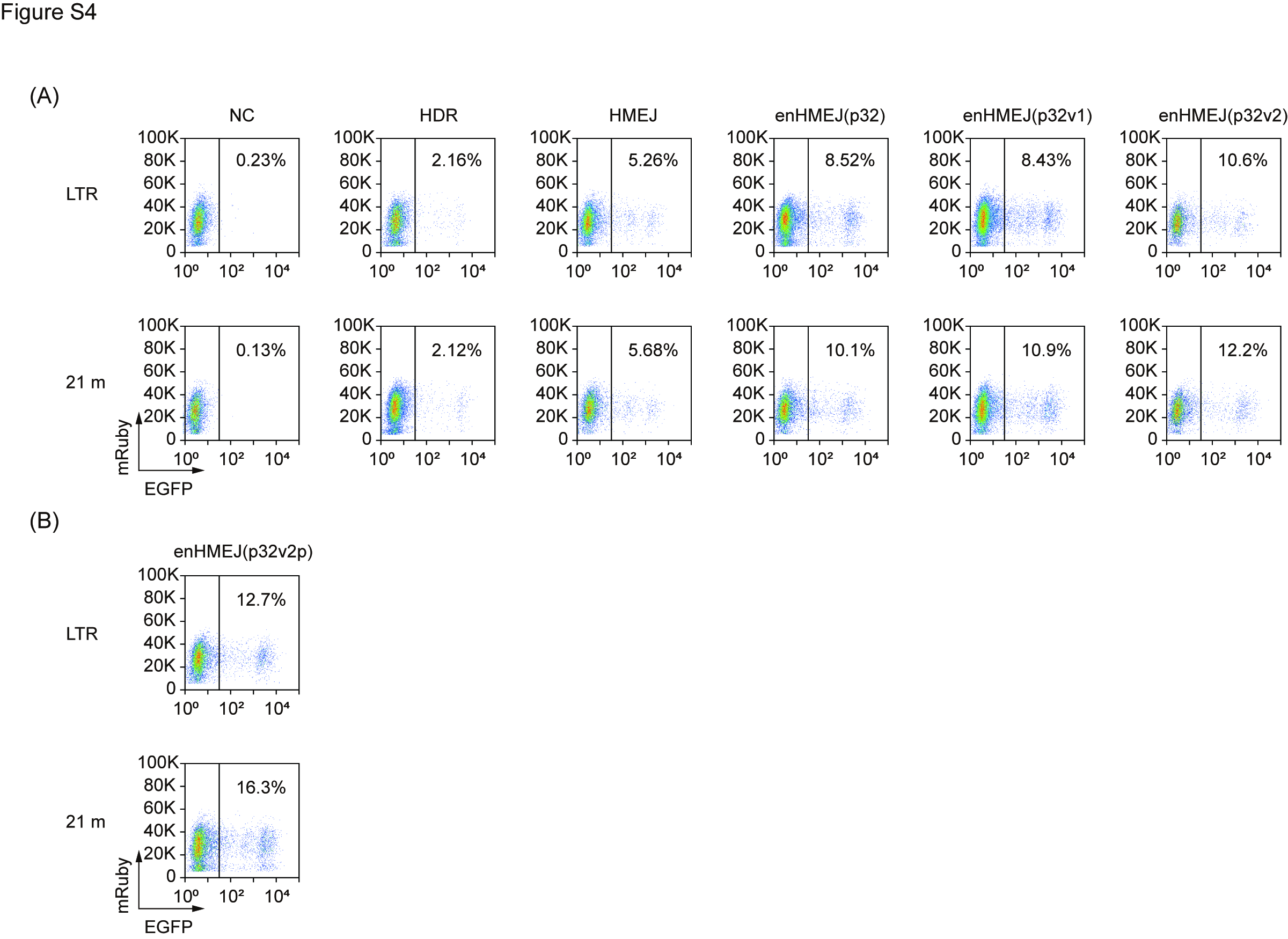


FIGURE S4

FACS analysis showing the percentage of EGFP^+^ cell. (A and B) FACS analysis showing of percentage of cell population that were EGFP^+^ based on different methods.


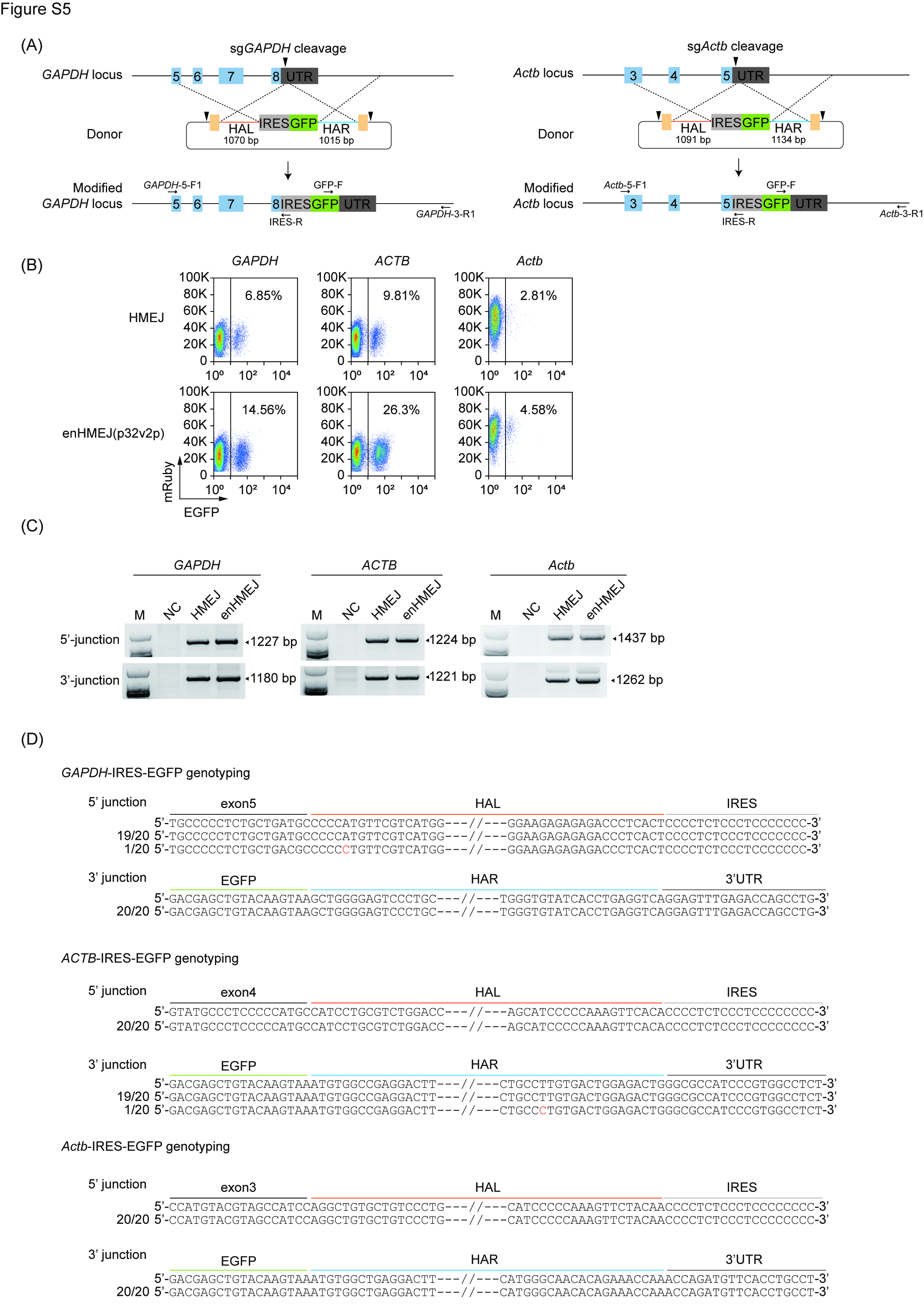


FIGURE S5

Targeted integration based on HMEJ and enHMEJ (p32v2p/21 m) in HEK293 and Hepa1-6 cell lines. (A) Schematics of targeted integration at the *GAPDH* loci in HEK293T cells and at the *Actb* loci in Hepa1-6 cells. (B) FACS analysis of the percentage of EGFP^+^ cell population from targeted integration at the *GAPDH*, *ACTB*, and *Actb* loci. (C) Genotyping analysis of the targeted mixed cells. (D) Sequencing analysis at the 5′-junction and 3′-junction of targeted *GAPDH*, *ACTB*, and *Actb* loci. Red text indicates the mutated or inserted bases.


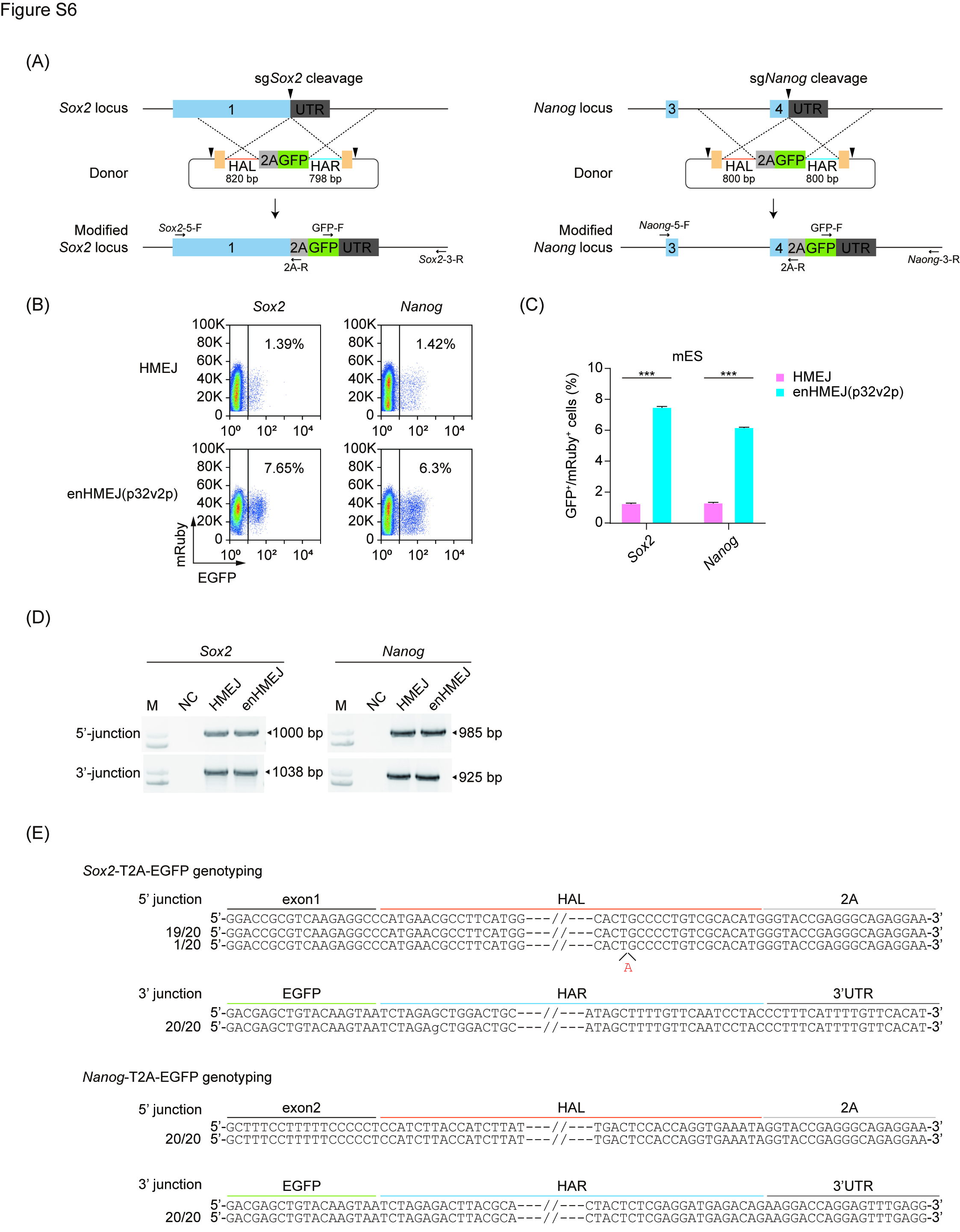


FIGURE S6

Targeted integration based on HMEJ and enHMEJ (p32v2p/21 m) in mouse embryonic stem cell (mES). (A) Schematics of targeted integration at *Sox2* and *Nanog* loci. (B and C) FACS analysis and statistics of percentage of EGFP^+^ cell population from targeted integration at the *Sox2* and *Nanog* loci. ****P* < 0.001 (unpaired Student’s *t*-test). (D) Genomic PCR of a targeted cell mixture generated by different methods. (E) Sequencing analysis at the 5′-junction and 3′-junction of the targeted *Sox2* and *Nanog* loci. Red text indicates the mutated or inserted bases.


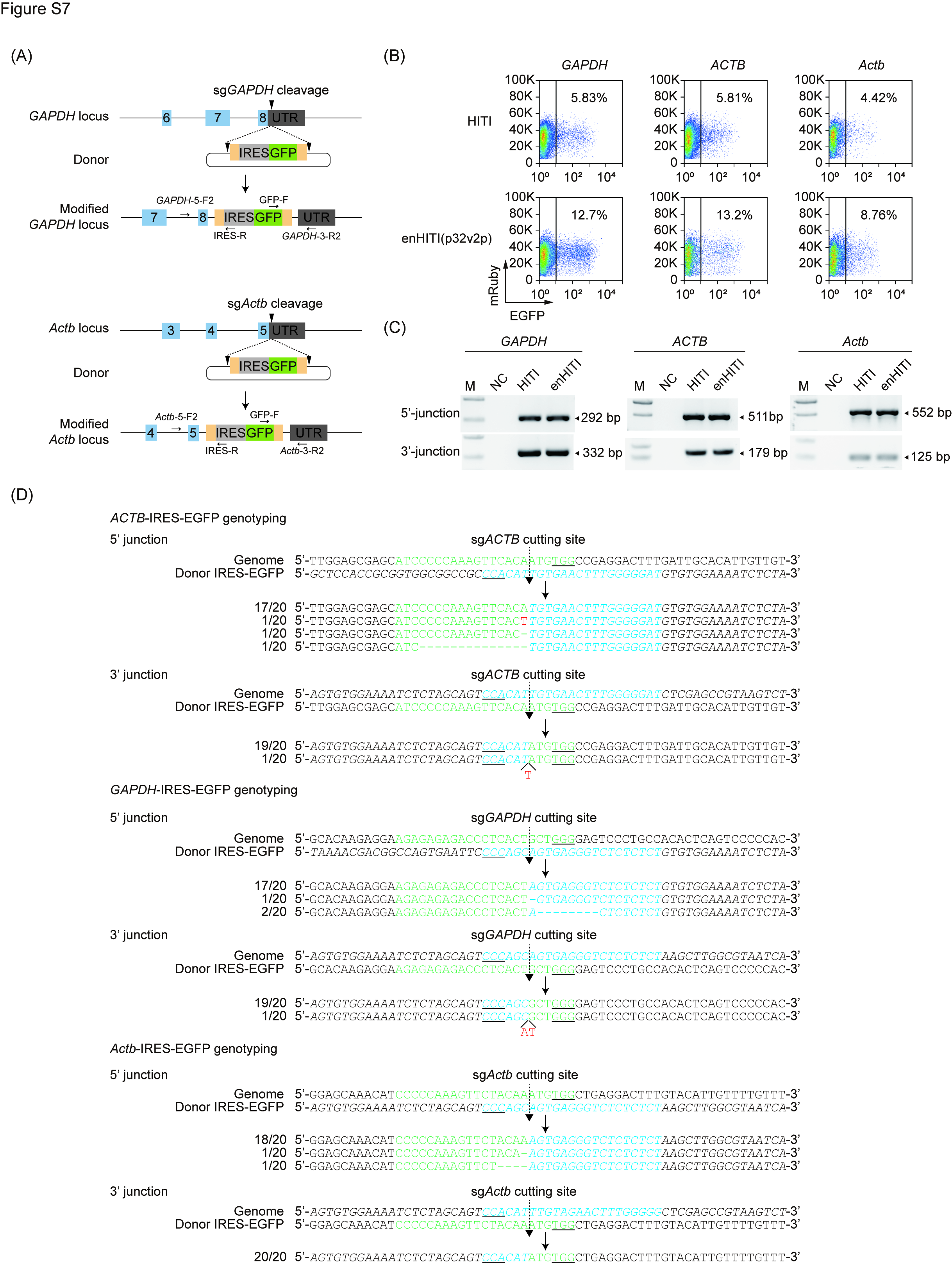


FIGURE S7

Targeted integration based on HITI and enHITI (p32v2p/21 m) in HEK293 and Hepa1-6 cell lines. (A) Schematics of targeted integration at the *GAPDH* and *Actb* loci. (B) FACS analysis results of the percentage of EGFP^+^ cell population from targeted integration at the *GAPDH*, *ACTB*, and *Actb* loci. (C) Genomic PCR of a targeted cell mixture generated by different methods. (D) Sequencing analysis at the 5′-junction and 3′-junction of targeted *GAPDH*, *ACTB*, and *Actb* loci. Green sequences indicate the Cas9/sgRNA target sequence on the genome, blue italic sequences indicate the Cas9/sgRNA target sequence on the donor, black underlining indicates the protospacer adjacent motif (PAM) of sgRNA, red text indicates the mutated or inserted bases, and dashed lines indicate the deleted bases after integration.

**Materials and Methods**

**Plasmid construction**

To mark cells transfected with Cas9, the red fluorescence protein mRuby2, which is acid insensitive, bright, has low cell toxicity, and exhibits excitation and emission spectra at 561 nm and 605 nm, respectively, was inserted into the modified Cas9 expression vector pX330-Cas9-CMV-EGFP (Addgene, 42230) to replace EGFP. The EGFP fragment in plasmid pX330 was removed by restriction endonucleases *Nhe*I-HF (NEB, R3131) and *Bam*HI-HF (NEB, R3136), and the vector was purified using a Zymoclean Gel DNA Recovery kit (Zymo Research, D4001). The mRuby2 fragment (708 bp) was amplified from the pET-mRuby2-LIC cloning vector (Addgene, 86932) with KOD One Master Mix-Blue (Toyobo, KMM-201) and was inserted into the linearized pX330 vector using NEBuilder HiFi DNA Assembly Master Mix (NEB, E2621). To generate the p32/v1/v2/v2p-Cas9 fused protein expression vector, fragments of the integrase p32 and its variants were amplified from psPAX2 (Addgene, 12260) and inserted into the linearized plasmid pX330-Cas9-CMV-mRuby2, which was digested with *Age*I-HF (NEB, R3552); the cleavage site for this restriction enzyme is located in front of Cas9 in the pX330-Cas9-CMV-mRuby2 plasmid. The sequence and length of p32 and its variants in this experiment are shown in Supplementary Table S3.

To generate sgRNA constructs, the pUC19-U6-sgRNA backbone was digested with *Bsa*I-HFv2 (NEB, R3733) so that 5′-CCGG and 5′-GTTT overhangs remained to accept the annealed oligos. The sgRNA target sequences are listed in Supplementary Table S1.

All donor vectors were constructed on a pPNT6 (Addgene, 11072) backbone. The plasmid pPNT6 was linearized with *Not*I-HF (NEB, R3189) and *Xho*I (NEB, R0146). To construct the donor pPNT6-LTR-GIP, the LTR fragment and the GFP fragment without an initiation codon, amplified from pHIV-EGFP (Addgene, 21373), were inserted into the linearized pPNT6 backbone. All the target donors used in HMEJ at genomic loci (*ACTB*, *GAPDH*, *Actb*, *Sox2*, and *Nanog*) were constructed by inserting the left homologous arm, IRES/2A-EGFP, and right homologous arms into the linearized pPNT6 plasmid by one-step cloning. The homologous arms were beside the 21 m LTR and site-specific sgRNA sequences. All target donors used in HITI at genome loci (*ACTB*, *GAPDH*, and *Actb*) were constructed by inserting the IRES-EGFP fragment, alongside the 21 m LTR and site-specific reversed sgRNA sequences, into the linearized pPNT6 plasmid. The sequences of LTR and 21 m LTR are shown in Supplementary Table S4.

**Cell culture**

All cells were incubated at 37°C with 5% CO_2_. HEK293T, 293T-EGIP and Hepa1-6 cells were cultured in Dulbecco’s modified Eagle’s medium (DMEM, Thermo Fisher Scientific, C11995500BT) supplemented with 10% fetal bovine serum (Thermo Fisher Scientific, 10099).

The mouse embryonic stem (mES) cell line was cultured in N2B27 medium supplemented with 1 µM PD0325901 (Stemgent), 3 µM CHIR99021 (Stemgent), and 1000 U/mL mLIF (Millipore). N2B27 medium was composed of DMEM/Nutrient Mixture F-12 (Gibco) and Neurobasal (Gibco) in a ratio of 1:1, and was supplemented with 1% N-2 supplement (Gibco), 0.5% B-27 supplement (Gibco), 10 µg/mL insulin (Roche Applied Science), 1% GlutaMAX (Gibco), 20 ng/mL bovine serum albumin (BSA, Sigma), 5% knockout serum replacement (KOSR, Gibco), 0.1% 2-mercaptoethanol (Gibco), and 1% antibiotic–antimycotic (Gibco).

**Cell transfection**

HEK293T, 293T-EGIP and Hepa1-6 cells were seeded in 6-well plates. One day after plating, when the seeded cells had grown to 80%-90% confluence, HEK293, 293T-EGIP and Hepa1-6 cells were transfected with a total of 4 µg plasmid mixture (Cas9:donor:sgRNA = 2:2:1) using Lipofectamine 3000 transfection reagent (Thermo Fisher Scientific, L3000015).

mES cells seeded in 6-well plates were digested to single cells with 0.25% trypsin-EDTA (Thermo Fisher Scientific; 25200056) and washed with phosphate-buffered saline (PBS). Then, 1×10^5^ mES cells were electroporated with a total of 10 µg plasmids mixture (Cas9:donor:sgRNA = 2:2:1).

**Flow cytometry**

Forty-eight hours after transfection, mRuby^+^ cells were sorted into 12-well plates by BD FACS. Five days later, the cultured cells were sorted again by FACS to analyze the percentage of GFP^+^ cells.

**Sanger sequencing analysis for genome-targeted modifications**

At the time of the second cell sorting, genomic DNA of the targeted cells was collected via extraction with 100 µL lysis buffer from the Mouse Directed PCR kit (Bimake, B40015). The targeted region was verified by PCR amplification with the appropriate primer pairs (Supplementary Table S2) using 2× Taq Master Mix (Vazyme, P112-01). The resulting PCR products were identified on a 1% agarose gel and cloned into a TA-cloning vector (Takara, 6011). The cloned vectors were transformed into a competent *Escherichia coli*. After culture on agar plates 16 h at 37°C, numerous clones were randomly selected and sequenced. Primer sequences used in this experiment are shown in Supplementary Table S2.

**ChIP-qPCR**

Three 15 cm dishes of HEK293T cell at 80%-90% confluence were prepared. Three groups comprising p32-Cas9 plus LTR-GOI-LTR, p32-Cas9 plus GOI, and Cas9 plus LTR-GOI-LTR were set in the experiment. Each dish was transfected with a total of 20 µg plasmids mixtures in a molar ratio of 1:1. The constructs p32-Cas9 plus GOI and Cas9 plus LTR-GOI-LTR were used as negative controls. Forty-eight hours after transfection, the cells were harvested according to the ChIP procedures (Cell Signaling Technology, 56383). A specifical Flag monoclonal antibody (Cell Signaling Technology, 14793) was used in the immunoprecipitation (IP) reaction. Purified DNAs were detected by qPCR with the standard qPCR kit (Toyobo, QPS-201). Primer sequences used in this experiment are shown in Supplementary Table S2.

**Supplementary Tables**

**Table S1. Sequence of sgRNA.**

| Name | Sequence |
| --- | --- |
| sgGFP | CAGCCACAACGTCTATATCATGG |
| sgACTB | ATCCCCCAAAGTTCACAATGTGG |
| sgGAPDH | AGAGAGAGACCCTCACTGCTGGG |
| sgActb | CCCCCAAAGTTCTACAAATGTGG |
| sgSox2 | TGCCCCTGTCGCACATGTGAGGG |
| sgNanog | CGTAAGTCTCATATTTCACCTGG |

**Table S2. Sequence of PCR primers.**

| Name | Sequence |
| --- | --- |
| KI-F | GAGCGCACGATTTTTTTTAAAGATG |
| KI-R | CTGATAGGCAGCCTGCACCTGAG |
| ACTB-5-F1 | CATGTACGTTGCTATCCAGGCTGTG |
| ACTB-3-R1 | GAGGTCCAGGAGCCTGGGTATCTC |
| GAPDH-5-F1 | ACTGTCTGCTTCTCTGCTGTAGGCTC |
| GAPDH-3-R1 | GGACTATAGGCATGTGCCATGAAGC |
| Actb-5-F1 | CTGAACTTAGCTGTGTTCTTGCACTCC |
| Actb-3-R1 | AATGCTCAATGTACAGTGATACTGGACAC |
| IRES-R | TAACATATAGACAAACGCACACCGG |
| GFP-F | ATCACATGGTCCTGCTGGAGTTCG |
| Sox2-5-F | GCATGTATAACATGATGGAGACGGAG |
| Sox2-3-R | CAGCCTGATTCCAATAACAGAGCC |
| Nanog-5-F | ATGTCCTGTCACTCTGCAGGTTAAGAC |
| Nanog-3-R | GCCTTGAACTTATTGCATATCTGAGC |
| 2A-R | CACGTCACCGCATGTTAGAAGACTTC |
| ACTB-5-F2 | GAGCTGTCACATCCAGGGTCCTC |
| ACTB-3-R2 | GGACTTCCTGTAACAACGCATCTCA |
| GAPDH-5-F2 | TGGTGGCTGGCTCAGAAAAAGG |
| GAPDH-3-R2 | GCAAGAATGTCTCACCTTGACACAAG |
| Actb-5-F2 | GAGAGCTCACCATTCACCATCTTGTC |
| Actb-3-R2 | AACAATGTACAAAGTCCTCAGCCACAT |
| ChIP-q-F | GTCGACAGCGTTTAAACGAGGTACC |
| ChIP-q-R | ACTTCAGGGTCAGCTTGCCGTAG |

**Table S3. Sequence of integrase and its variants.**

| Name | Sequence |
| --- | --- |
| p32  (783 bp) | GCCCAAGAAGAACATGAGAAATATCACAGTAATTGGAGAGCAATGGCTAGTGATTTTAACCTACCACCTGTAGTAGCAAAAGAAATAGTAGCCAGCTGTGATAAATGTCAGCTAAAAGGGGAAGCCATGCATGGACAAGTAGACTGTAGCCCAGGAATATGGCAGCTAGATTGTACACATTTAGAAGGAAAAGTTATCTTGGTAGCAGTTCATGTAGCCAGTGGATATATAGAAGCAGAAGTAATTCCAGCAGAGACAGGGCAAGAAACAGCATACTTCCTCTTAAAATTAGCAGGAAGATGGCCAGTAAAAACAGTACATACAGACAATGGCAGCAATTTCACCAGTACTACAGTTAAGGCCGCCTGTTGGTGGGCGGGGATCAAGCAGGAATTTGGCATTCCCTACAATCCCCAAAGTCAAGGAGTAATAGAATCTATGAATAAAGAATTAAAGAAAATTATAGGACAGGTAAGAGATCAGGCTGAACATCTTAAGACAGCAGTACAAATGGCAGTATTCATCCACAATTTTAAAAGAAAAGGGGGGATTGGGGGGTACAGTGCAGGGGAAAGAATAGTAGACATAATAGCAACAGACATACAAACTAAAGAATTACAAAAACAAATTACAAAAATTCAAAATTTTCGGGTTTATTACAGGGACAGCAGAGATCCAGTTTGGAAAGGACCAGCAAAGCTCCTCTGGAAAGGTGAAGGGGCAGTAGTAATACAAGATAATAGTGACATAAAAGTAGTGCCAAGAAGAAAAGCAAAGATCATCAGGGATTATGGAAAACAGATGGCAGGTGATGATTGTGTGGCAAGTAGACAGGATGAGGAT |
| v1  (660 bp) | GGAATATGGCAGCTAGATTGTACACATTTAGAAGGAAAAGTTATCTTGGTAGCAGTTCATGTAGCCAGTGGATATATAGAAGCAGAAGTAATTCCAGCAGAGACAGGGCAAGAAACAGCATACTTCCTCTTAAAATTAGCAGGAAGATGGCCAGTAAAAACAGTACATACAGACAATGGCAGCAATTTCACCAGTACTACAGTTAAGGCCGCCTGTTGGTGGGCGGGGATCAAGCAGGAATTTGGCATTCCCTGCAATCCCCAAAGTCAAGGAGTAATAGAATCTATGAATAAAGAATTAAAGTGTATTATAGGACAGGTAAGAGATCAGGCTGAACATCTTAAGACAGCAGTACAAATGGCAGTATTCATCCACAATTTTAAAAGAAAAGGGGGGATTGGGGGGTACAGTGCAGGGGAAAGAATAGTAGACATAATAGCAACAGACATACAAACTAAAGAATTACAAAAACAAATTACAAAAATTCAAAATTTTCGGGTTTATTACAGGGACAGCAGAGATCCAGTTTGGAAAGGACCAGCAAAGCTCCTCTGGAAAGGTGAAGGGGCAGTAGTAATACAAGATAATAGTGACATAAAAGTAGTGCCAAGAAGAAAAGCAAAGATCATCAGGGATTATGGAAAACAGATGGCAGGTGAT |
| v2  (486 bp) | GGAATATGGCAGCTAGATTGTACACATTTAGAAGGAAAAGTTATCTTGGTAGCAGTTCATGTAGCCAGTGGATATATAGAAGCAGAAGTAATTCCAGCAGAGACAGGGCAAGAAACAGCATACTTCCTCTTAAAATTAGCAGGAAGATGGCCAGTAAAAACAGTACATACAGACAATGGCAGCAATTTCACCAGTACTACAGTTAAGGCCGCCTGTTGGTGGGCGGGGATCAAGCAGGAATTTGGCATTCCCTACAATCCCCAAAGTCAAGGAGTAATAGAATCTATGAATAAAGAATTAAAGAAAATTATAGGACAGGTAAGAGATCAGGCTGAACATCTTAAGACAGCAGTACAAATGGCAGTATTCATCCACAATTTTAAAAGAAAAGGGGGGATTGGGGGGTACAGTGCAGGGGAAAGAATAGTAGACATAATAGCAACAGACATACAAACTAAAGAATTACAAAAACAAATTACAAAAATT |
| v2p  (972 bp) | GGAATATGGCAGCTAGATTGTACACATTTAGAAGGAAAAGTTATCTTGGTAGCAGTTCATGTAGCCAGTGGATATATAGAAGCAGAAGTAATTCCAGCAGAGACAGGGCAAGAAACAGCATACTTCCTCTTAAAATTAGCAGGAAGATGGCCAGTAAAAACAGTACATACAGACAATGGCAGCAATTTCACCAGTACTACAGTTAAGGCCGCCTGTTGGTGGGCGGGGATCAAGCAGGAATTTGGCATTCCCTACAATCCCCAAAGTCAAGGAGTAATAGAATCTATGAATAAAGAATTAAAGAAAATTATAGGACAGGTAAGAGATCAGGCTGAACATCTTAAGACAGCAGTACAAATGGCAGTATTCATCCACAATTTTAAAAGAAAAGGGGGGATTGGGGGGTACAGTGCAGGGGAAAGAATAGTAGACATAATAGCAACAGACATACAAACTAAAGAATTACAAAAACAAATTACAAAAATTGGAATATGGCAGCTAGATTGTACACATTTAGAAGGAAAAGTTATCTTGGTAGCAGTTCATGTAGCCAGTGGATATATAGAAGCAGAAGTAATTCCAGCAGAGACAGGGCAAGAAACAGCATACTTCCTCTTAAAATTAGCAGGAAGATGGCCAGTAAAAACAGTACATACAGACAATGGCAGCAATTTCACCAGTACTACAGTTAAGGCCGCCTGTTGGTGGGCGGGGATCAAGCAGGAATTTGGCATTCCCTACAATCCCCAAAGTCAAGGAGTAATAGAATCTATGAATAAAGAATTAAAGAAAATTATAGGACAGGTAAGAGATCAGGCTGAACATCTTAAGACAGCAGTACAAATGGCAGTATTCATCCACAATTTTAAAAGAAAAGGGGGGATTGGGGGGTACAGTGCAGGGGAAAGAATAGTAGACATAATAGCAACAGACATACAAACTAAAGAATTACAAAAACAAATTACAAAAATT |

**Table S4. Sequence of LTR and 21 m LTR.**

| Name | Sequence |
| --- | --- |
| LTR | GGGTCTCTCTGGTTAGACCAGATCTGAGCCTGGGAGCTCTCTGGCTAACTAGGGAACCCACTGCTTAAGCCTCAATAAAGCTTGCCTTGAGTGCTTCAAGTAGTGTGTGCCCGTCTGTTGTGTGACTCTGGTAACTAGAGATCCCTCAGACCCTTTTAGTCAGTGTGGAAAATCTCTAGCA |
| 21 m LTR | GTGTGGAAAATCTCTAGCAGT |
